# The incidental discovery of a constitutional trisomy 21 mosaicism in an adult female with myelodysplastic/myeloproliferative neoplasm

**DOI:** 10.1007/s00277-021-04655-0

**Published:** 2021-09-01

**Authors:** Samantha O’Hagan Henderson, Anita Glaser, Jochen J. Frietsch, Andreas Hochhaus, Inken Hilgendorf

**Affiliations:** 1 Klinik für Innere Medizin II - Onkologie und Hämatologie, Universitätsklinikum Oldenburg, Oldenburg, Germany; 2 Institut für Humangenetik, Universitätsklinikum Jena, Jena, Germany; 3 Klinik für Innere Medizin II, Abteilung für Hämatologie und Internistische Onkologie, Universitätsklinikum Jena, Am Klinikum 1, 07747 Jena, Germany

Dear Editor,

Here we report the case of an adult with myelodysplastic syndrome/myeloproliferative neoplasm (MDS/MPN) and constitutional trisomy 21 mosaicism as the only identified chromosomal abnormality.

A 44-year-old female patient with weight loss, night sweats, and fatigue was diagnosed with MPN (bone marrow (BM) fibrosis (I^o^) and splenomegaly). *GATA1*, *BCR-ABL*, *MPL*, *CALR*, and *JAK2* mutations were negative. Upon cytogenetic examination of the malignant cells, 30% were found to have trisomy 21. This molecular mosaicism was also found in buccal mucosa cells, showing that the patient was a constitutional trisomy 21 mosaic.

A year after the diagnosis, BM biopsy revealed transformation to MDS RAEB II. An allogeneic stem cell transplantation was carried out. Three months later, the disease relapsed. Despite a stem cell boost and reduction of immunosuppression, she died on day + 226 from sepsis.

The presence of constitutional trisomy 21 mosaicism may have been significant in the development of the hematological malignancy. Individuals with trisomy 21 mosaicism have two genetically distinct cell lines that develop from a single zygote. Their bodies contain both trisomic and euploid cells. Their phenotypes vary widely, from those similar to Down syndrome (DS) to those with no signs or symptoms at all.

DS is associated with an increased risk of MDS or leukemia in children [1]. Almost all cases of acute megakaryoblastic leukemia (AMKL) in patients under the age of four are associated with a *GATA-binding factor 1* loss-of-function mutation. *GATA1* plays an important role in the expression of genes mediating the maturation of erythro- and thrombocytes. Trisomy 21 is considered the first genetic event in the development of AMKL with the *GATA1* mutation being the second hit required for leukemogenesis.

Although acquired trisomy 21 has been identified as one of the most common abnormalities in AML, MDS, and MPN, present in between 4 and 7% of cases [2], it does not often occur as the sole numerical karyotypic abnormality [3]. The presence of the extra chromosome appears to lead to genome-wide hypomethylation of histones [4]. In addition, a number of genes on chromosome 21 have been implicated in the development of AML, e.g., *RUNX1* (runt-related transcription factor 1) [5].

While children with DS and AMKL, over the age of four, have a reduced sensitivity to chemotherapy and poor prognosis [6], the hematological fate of adults with DS is poorly defined. Compared to healthy controls, they had hematological abnormalities, e.g., a higher mean cell volume or unexplained neutropenia [7]. This raises the possibility that a greater proportion of adults with DS have undiagnosed myelodysplasia or BM failure than previously appreciated and that trisomy 21 perturbs normal hematopoiesis throughout life [5]. In the most widely used mouse model for DS, MPN manifests in adult, rather than fetal mice [8].

Although AML with a *GATA1* mutation has been identified in children with trisomy 21 mosaicism [9], this is to our knowledge, the first reported case of an adult without *GATA1* mutation and with an MDS/MPN and constitutional trisomy 21 mosaicism as the only identified chromosomal abnormality.
